# Psychosocial predictors of hereditary cancer genetic testing motivation in untested individuals

**DOI:** 10.1002/jgc4.70122

**Published:** 2025-10-15

**Authors:** Sarah Austin, Emerson Delacroix, John D. Rice, Erika Koeppe, Elena M. Stoffel, Jennifer J. Griggs, Kenneth Resnicow

**Affiliations:** ^1^ Department of Internal Medicine, School of Medicine University of Michigan Ann Arbor Michigan USA; ^2^ Rogel Cancer Center University of Michigan Ann Arbor Michigan USA; ^3^ School of Public Health University of Michigan Ann Arbor Michigan USA; ^4^ School of Public Health University of Minnesota Minneapolis Minnesota USA

**Keywords:** hereditary cancer, genetic testing, healthcare distrust

## Abstract

Genetic testing for hereditary cancer syndromes can provide lifesaving information allowing for individualized cancer screening, prevention, and treatment. A broader understanding of how psychosocial factors impact motivation to undergo genetic testing is needed to improve uptake among individuals who would benefit from testing. Adults (≥18 years) who met criteria for genetic testing based on a self‐reported family cancer history and had not previously completed testing were invited to complete a survey (*n* = 799) assessing psychosocial factors including barriers to genetic testing, healthcare distrust, perceived self‐efficacy, clinician autonomy support, and genetic testing knowledge. Associations between these psychosocial factors and testing motivation were examined first by correlation followed by multivariable linear regression. Self‐efficacy had a significant positive correlation with genetic testing motivation, while barriers and healthcare distrust were negatively correlated with motivation. In an adjusted multivariable regression model, higher self‐efficacy was associated with higher motivation while higher barriers and healthcare distrust were negatively associated with genetic testing motivation. Individuals of older age (51+), non‐White race, and lower perceived socioeconomic status reported higher mean motivation scores. The negative association between distrust and barriers with genetic testing intention may be a potential target for tailored interventions for genetic testing.

**What is known about this topic**

Several factors impacting genetic testing motivation have been identified, but there is little research specific to the role healthcare distrust has on genetic testing motivation.

**What this paper adds to the topic**

Multivariable analysis identified a set of psychosocial variables including healthcare distrust, self‐efficacy, and barriers, as well as demographic variables including age as potential items necessitating intervention for patients meeting genetic testing criteria due to either personal and/or family history of cancer.

## 1 INTRODUCTION

Germline pathogenic variants associated with increased cancer risk are implicated in 5–20% of all cancers (Kurian et al., 2023; Robinson et al., 2017). Identification of these genetic variants impacts cancer screening, prevention, treatment, and surveillance for affected individuals and their blood relatives. Despite the importance of testing for clinical care, only a fraction of people at risk for hereditary cancer syndromes undergo genetic testing, with a recent study showing that only 6.8% of patients with cancer have been tested (Kurian et al., 2023).

Several factors impact genetic testing decisions for at‐risk individuals, including having a physician referral/recommendation, discussing genetic testing with a healthcare provider, and logistical challenges (Delacroix et al., 2023; Hafertepen et al., 2017; Ladd et al., 2020; Loeb et al., 2021; Saylor et al., 2024; Willis et al., 2017). Psychosocial factors, such as cancer distress, individual perception of cancer risk, genetics knowledge, stigma and concerns about stigma, and self‐efficacy have also been shown to affect genetic testing intention and uptake (Austin et al., 2024; Chang et al., 2022; Sweeny et al., 2014). A systematic review from 2021 assessed motivation to pursue genomic testing for those with a personal and/or family history of cancer and found that interest in predicting cancer occurrence/recurrence, cancer management and clinical utility, impact on relatives, cost, and privacy/confidentiality all played a role in testing decisions (Smith‐Uffen et al., 2021). This study, and our previous work, has shown that concern about family members and the clinical utility of testing can serve as both barriers and motivating factors to pursuing testing (Austin et al., 2024; Smith‐Uffen et al., 2021). Similarly, patients with low or limited genetics knowledge related to cancer risk were still interested in, and often pursued, genetic testing, suggesting there are other factors impacting one's interest and ability to get tested (Chapman et al., 2019; Gomez‐Trillos et al., 2020; Sussner et al., 2011). The contribution of medical distrust/mistrust as potential determinants of genetic testing uptake has received limited attention and was not assessed in this previous work.

Medical distrust and mistrust have been shown to impact a variety of health behaviors, including vaccination compliance, HIV prevention, chronic disease management, and in particular, cancer screening (Bartolome et al., 2016; Charura et al., 2023; Miriyala et al., 2025; Nipher et al., 2024; Ponce et al., 2025; Relf et al., 2019). While both mistrust and distrust are rooted in lack of trust, there are distinctions between the two terms. Some researchers define distrust as the belief that an individual or institution is acting against one's best interest based on their personal knowledge and experience, whereas mistrust has been defined as a general sense of suspicion that may originate from historical experiences and discrimination rather than from personal interactions (Griffith et al., 2021; Mouslim et al., 2020). These distinctions are not universally used; however, in this current study, our measures relate more to distrust as defined above, rather than mistrust.

There is limited literature specific to healthcare distrust and genetic testing. One study found higher distrust, measured through the revised Health Care System Distrust Scale (HCSDS), was related to lower willingness to undergo genetic testing (Armstrong et al., 2012; Shea et al., 2008). Other studies measuring distrust include Alvord et al. (2020), who completed focus groups with Oregon residents from multiple communities to gather information on perceptions of genetic testing and interest in participating in genetics research. Distrust, among other concerns, was reported by participants at 80% of the focus group sites and was reported similarly across urban, rural, and frontier communities. Participants reported distrust with research institutions, science, and insurance companies as well as concern with the accuracy of genetic testing, suggesting both general and specific distrust in the healthcare system (Alvord et al., 2020). The relationship between healthcare distrust and preventive care is substantiated in a systematic review by Mouslim et al. (2020). Authors identified four studies related to breast cancer screening and healthcare distrust, which used the HCSDS or revised HCSDS, and found a significant negative relationship between distrust and screening outcomes (lower odds of having a clinical breast exam, receiving cancer screening in the past 2 years, and lower satisfaction with mammography services; Mouslim et al., 2020).

The relationship between mistrust and genetic testing has also been examined in a few studies. In ethnic minority populations, individuals have reported concerns about potential misuse of genetic data and a belief that testing was offered for profit rather than medical necessity (Hann et al., 2017). Sutton et al. (2019) surveyed 94 Black women at risk for hereditary breast and ovarian cancer and found that those who experienced more discrimination and had less confidence in their ability to obtain genetic testing had greater medical mistrust (Sutton et al., 2019). In another study of Black and African American oncology patients, higher levels of medical mistrust, assessed using the Group‐Based Medical Mistrust scale, were associated with greater concern about genetic profiling than those with lower medical mistrust (Hoadley et al., 2022). Finally, a systematic review revealed two studies assessing mistrust related to breast cancer genetic testing using the Medical Mistrust Index and Group‐Based Medical Mistrust Scale in White, Black, and Latina adult women. In one study, higher mistrust was related to lower genetic testing/counseling engagement, and the other found higher mistrust was related to more concerns and perceived disadvantages about breast cancer genetic testing (Mouslim et al., 2020). These studies mainly focused on mistrust across racial and/or ethnic minority populations rather than distrust of the healthcare system.

A broader understanding of how psychosocial factors, including healthcare distrust, impacts genetic testing decision making is needed. The aim of this study is to examine how certain social‐cognitive determinants and barriers relate to motivation to undergo hereditary cancer genetic testing among patients who have not been previously tested.

## 2 METHODS

### 2.1 Participants

As part of a large clinical trial evaluating interventions to promote uptake of genetic testing for cancer susceptibility in previously untested individuals, adults were invited to complete a family health history survey eliciting detailed cancer history for individuals and their first and second degree biological relatives (Gerido et al., 2023, ClinicalTrials.gov NCT05162846). Sites of subject recruitment included community oncology practices in the state of Michigan, cancer registries, oncology, gastroenterology, and primary care clinics at an academic medical center, community health fairs, and radio and newspaper advertisements. The University of Michigan Medical School Institutional Review Board approved this research.

Individuals meeting clinical guidelines for genetic testing based on family or personal history of cancer received an email invitation from the study team with a unique link to complete a brief survey to assess eligibility for the clinical trial. Individuals who had already completed genetic testing or had a genetic testing appointment scheduled, did not have access to a phone or internet, were under age 18, did not communicate in English, or were deceased were excluded from the trial.

Eligible and consented subjects were asked to complete a baseline survey that included questions about hereditary cancer knowledge, experience with genetic testing, and barriers and motivators to genetic testing. Reminder emails to complete the baseline survey were sent at 1, 3, 5, and 10 days post‐consent. Initially, up to eight, later five, reminder phone calls were made one to three times each week for 4 weeks.

### 2.2 Instrumentation

Participants were asked to respond to psychosocial measures in the baseline survey, including barriers to genetic testing, healthcare distrust, self‐efficacy, genetics knowledge, clinician autonomy support, and motivation to get genetic testing. Barrier and self‐efficacy items were both derived from previously validated measures, as well as created de novo, while all other psychosocial items were derived from prior measures, as described in more detail below.

Barriers to genetic testing spanned a variety of themes including cost, family worry, insurance/genetic discrimination, stigma, logistic challenges, and knowledge barriers (sample item: *I am too overwhelmed to think about genetic testing right now*). Barrier items were identified through review of the literature, and when no suitable measure could be identified, novel measures were developed leveraging investigator expertise (Austin et al., 2024). Barriers were assessed by creating an aggregate index score (adding the number of 4 or higher scores) from 23 items answered along a 5‐point Likert scale (1 = strongly disagree, 5 = strongly agree), referred to as “barrier index score”. Individual barrier items, mean scores, and correlations with motivation are included in Table S1.

Healthcare distrust was assessed with the 10‐item HCSDS (Rose et al., 2004). As the scale measures both general attitudes (“*The health care system cares more about holding costs down than it does about doing what is needed for my health*”) and personal experiences (“*When they take my blood, they do tests they don't tell me about*”), we consider it a measure of both distrust and mistrust (Rose et al., 2004). All items were answered along a 5‐point Likert scale (1 = strongly disagree, 5 = strongly agree) computed into a mean score.

Self‐efficacy, that is, one's perceived ability to understand genetic information, was assessed through six items, five of which were adapted from a prior intervention and one created de novo (Kinney et al., 2018). Sample items include “I am confident I know what action to take based on my cancer genetic testing results” and “I am confident I know where and how to get tested.” Items were answered along a 7‐point Likert scale (1 = strongly disagree, 7 = strongly agree) and aggregated as a mean.

Genetic knowledge/literacy was assessed using the 16 true/false items from the KnowGene scale with the option of “I don't know,” which was coded as incorrect (Underhill‐Blazey et al., 2019). These items were converted to a number correct score. A sample item includes: *people with an inherited risk for cancer may get cancer at a younger age than people with average risk (Agree/Disagree)*.

Clinician autonomy support was assessed with seven items drawn from the Healthcare Climate Questionnaire (HCCQ) adapted to assess participants' relationship with “my primary cancer care provider” rather than “my doctor”, as in the original measure (Williams et al., 1996). All items were answered along a 7‐point Likert scale (1 = strongly disagree, 7 = strongly agree) and converted to a mean. A higher score is indicative of higher perceived autonomy support. These items were only answered by those who reported a personal cancer history and had a primary cancer care provider (sample item: *I feel my primary cancer care provider understands how I see things with respect to my cancer*).

Our primary dependent variable was motivation to get genetic testing, which was assessed with two items answered using a 10‐point Likert scale (1) “How ready are you to get genetic testing in the next year?” (0 = not at all ready, 10 = very ready) and (2) “How important to you is getting genetic testing in the next year?” (0 = not at all important, 10 = very important). Readiness and importance were aggregated to form a composite motivation score by taking the mean of the two variables.

The sociodemographic variables collected included gender identity (male, female, trans‐man, trans‐woman, non‐binary/genderqueer) which were collapsed into three levels: (1) male, (2) female, (3) non‐binary, genderqueer, or transgender. Current age was a discrete variable collapsed into three levels: ages 18–50, 51–70, ≥ 71. Education initially included 10 response options: no formal education, grade school, not high school graduate, high school graduate (including equivalency), some college/associate degree, vocational/technical school, bachelor's degree, master's degree, doctoral degree, and prefer not to answer, which were dichotomized into “less than a college degree” and “bachelor's degree or higher”, as consistent with prior literature (Skalamera & Hummer, 2016; Winkleby et al., 1992). Perceived financial stress included four choices: living comfortably on present income, getting by on present income, finding it difficult on present income, finding it very difficult on present income, which were dichotomized into living comfortably and getting by or finding it (very) difficult. Perceived financial stress was used rather than actual reported income as there were multiple missing responses for income.

Insurance status initially included eight response options: a plan purchased through an employer or union (including plans purchased through another person's employer); a plan that you or another family member buys on your own; Medicare; Medicaid or other state program; Tri‐care/Veterans Affairs, or Military; Alaska Native/Indian Health Service/Tribal Health Service; some other source; and none (no coverage), which were collapsed into two levels: public/government insurance/no insurance (combined due to low rate for none (no coverage)) and private insurance. Employment status comprised 12 response options (choose all that apply): full‐time job; part‐time job; doing paid work on a self‐employed basis or within your own business; “gig” worker/freelancer/consultant; student/on a government training program; out of work (6 months or less); out of work (more than 6 months); looking after home/homemaker; unpaid work for a business/community or voluntary organization; retired; and disabled/long‐term sick, which were collapsed into three levels: employed, unemployed/student, and retired/disabled.

Race included seven response options (choose all that apply): Native American or American Indian or Alaska Native, Asian or Asian American, Black or African American, Middle Eastern or North African, Native Hawaiian or other Pacific Islander, White or European American, and other. Ethnicity included two response options: Hispanic or Latino/a and not Hispanic or Latino/a. Race and ethnicity items were combined into a single variable for analyses, dichotomized into non‐Hispanic White vs. all other races and/or ethnicities given the low sample size for non‐White individuals.

The subjects' cancer history was trichotomized into a hierarchy. *Level 1* included personal history of (1) early age of onset (diagnosed at <50 years of age) breast, colorectal, endometrial/uterine, or prostate cancer or (2) personal history of ovarian or pancreatic cancer (diagnosed at any age). *Level 2* included any other personal history of any cancer meeting criteria for genetic evaluation (including Level 1 cancer types diagnosed at age ≥50). *Level 3* included individuals without a personal history of cancer who were eligible for genetic testing based on family history only.

The rationale for categorizing cancer histories this way is based on the fact that Level 1 cancers have been specifically highlighted in genetic testing guidelines for >10 years, and eligibility for genetic testing relies on personal history only. In contrast, Level 2 cancer histories include additional cancer types and/or cancers diagnosed at older ages, which, with the addition of family cancer history, would meet genetic testing criteria. Finally, individuals with Level 3 cancer history are identified solely by their family history of cancer, with no personal history of cancer.

### 2.3 Data analysis

Demographic variables were summarized by frequency. Associations between psychosocial items (barrier index score and mean scores from genetics knowledge/literacy, self‐efficacy, healthcare distrust, and clinician autonomy support items) with genetic testing motivation were assessed using pairwise Pearson correlation coefficients. A general linear regression model was used to investigate the relationship between psychosocial items (independent variables) and genetic testing motivation (dependent variable). The clinician autonomy support variable was removed from the final model given lower sample size, as only patients with a cancer care provider were shown that item in the survey. The model controlled for demographic variables previously described, including gender, age, education, perceived financial stress, insurance status, employment status, race, and cancer history. Data were analyzed using R and SPSS v.28. The dataset supporting this study is available at https://deepblue.lib.umich.edu/data/concern/data_sets/5m60qs76r.

## 3 RESULTS

**FIGURE 1:**
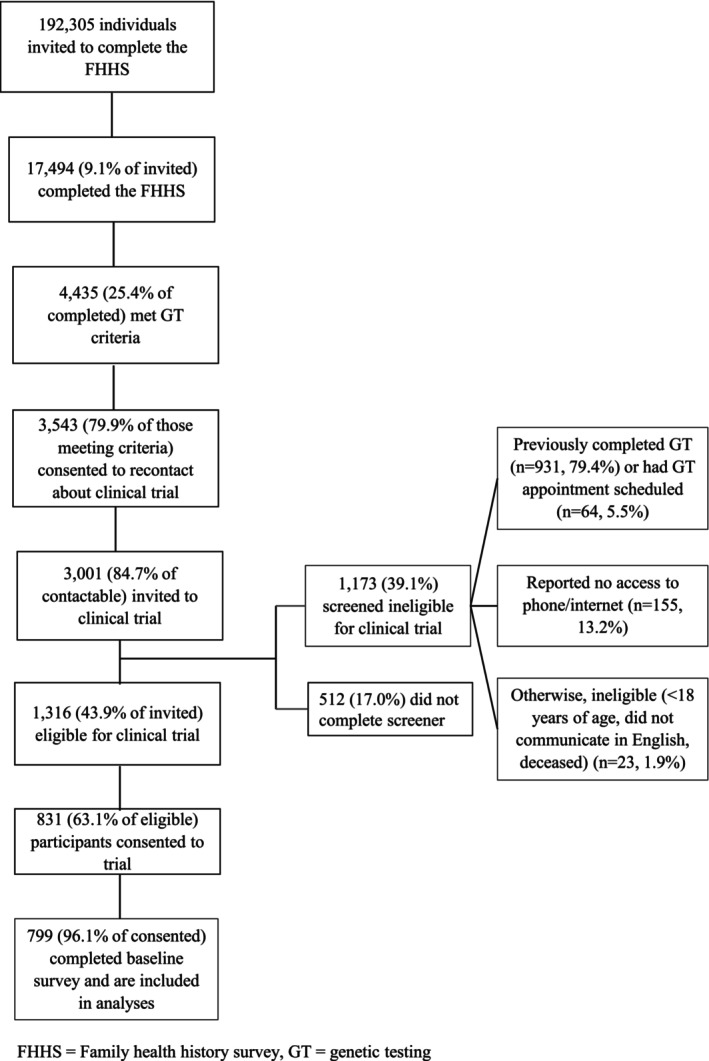
Flow diagram of participant enrollment.

An overview of study participant enrollment and dropout is outlined in Figure 1. 17,494 of 192,305 (9.1%) individuals invited completed the family health history survey and 3001 individuals who completed the survey met genetic testing criteria, consented to be contacted about the trial, and were invited to participate. 1173 (39.1%) of these invited individuals were found to be ineligible for the trial due to having already completed genetic testing (*n* = 931), having a genetic testing appointment scheduled (*n* = 64), not having access to a phone or internet (*n* = 155), being under age 18 (*n* = 1), being non‐English speaking (*n* = 7), or deceased (*n* = 15). 831 (27.7% of invited individuals) completed informed consent for the trial. Ultimately, 799 untested participants completed the baseline survey and were included in these analyses.

Demographic variables are summarized in Table 1. Of the 799 respondents, the sample was predominately female, White, and over 51 years of age. Most respondents had a bachelor's degree or higher, and over half of the sample reported living comfortably on their current income. Just over half of the participants had Level 2 cancers (51.6%).

**TABLE 1:** Participant characteristics (*N* = 799).

Demographics	*N* = 799
Gender	Count (%)
Female	466 (58.3%)
Male	324 (40.6%)
Non‐binary, Genderqueer or Transgender	8 (1.0%)
Missing	1 (0.1%)
Race and/or Ethnicity	
Other Race and/or Ethnicity (American Indian or Native American, Alaskan Native, Asian or Asian American, Black or African American, Hispanic/Latinx‐only, Middle Eastern or North African, Multiracial)	107 (13.4%)
White or European American, non‐Hispanic	664 (83.1%)
Missing	28 (3.5%)
Age (in years) at baseline	
18–50	199 (24.9%)
51–70	383 (47.9%)
≥71	217 (27.2%)
Education	
Less than a college degree (no formal education, grade school, not high school graduate, high school graduate, some college/associate degree, vocational/technical school)	242 (30.3%)
Bachelors degree or higher	555 (69.5%)
Missing	2 (0.3%)
Employment	
Currently employed (full or part time)	373 (46.7%)
Unemployed or student	77 (9.6%)
Retired or disabled	349 (43.7%)
Perceived Income/Financial Stress	
Getting by, Finding it difficult or very difficult	286 (35.8%)
Living comfortably	485 (60.7%)
Missing or prefer not to answer	28 (3.5%)
Health insurance type	
Public/government or none	370 (46.3%)
Private	401 (50.2%)
Missing	28 (3.5%)
Personal history of cancer	
Level 1 cancer history^a^	100 (12.5%)
Level 2 cancer history^a^	412 (51.6%)
No personal history of cancer (Level 3)	287 (35.9%)

*Note*: Descriptive statistics of baseline survey respondents.

^a^ Level 1 cancer includes early age of onset breast, colorectal, endometrial/uterine, or prostate cancer (diagnosed at <50 years of age), or a personal history of ovarian or pancreatic cancer (diagnosed at any age). Level 2 cancer history includes any cancer meeting criteria for genetic evaluation (including Level 1 cancer types diagnosed at age >50).

Internal consistency alpha was calculated for psychosocial items and motivation score and was over 0.70 for all items (Table S2).

### 3.1 Univariate analyses

Correlations between psychosocial factors and genetic testing motivation are shown in Table 2. Self‐efficacy had a significant positive correlation with motivation, while barrier index score and healthcare distrust had significant negative correlations with motivation (*p* < 0.001). Specifically, self‐efficacy was correlated at 0.16 with motivation, and barrier index score and healthcare distrust were negatively correlated at −0.14 and −0.17, respectively, with motivation. One‐way ANOVA showed a higher mean mistrust score for non‐White race and lower perceived income (*p* < 0.001).

**TABLE 2:** Pearson correlations of psychosocial factors to genetic testing motivation in untested individuals (*n* = 774).

Psychosocial variable	Pearson correlations with motivation	*p*
Self‐efficacy	0.16	<0.001
Genetic literacy/knowledge	0.06	0.09
Barrier index score	−0.14	<0.001
Distrust	−0.17	<0.001
Clinician autonomy support^a^	0.09	0.06

^a^ (*n* = 484) as only those with a personal cancer history were shown this question.

### 3.2 Multivariable analyses

In multivariable analysis, self‐efficacy was positively associated with motivation (*p* < 0.001), whereas barriers (*p* = 0.006) and healthcare distrust (*p* < 0.001) were inversely associated with motivation (Table 3); these models were adjusted for age, race/ethnicity, perceived income, gender, insurance, employment status, and cancer history. Individuals in the two oldest age groups had lower mean motivation scores compared with those age 50 and younger (*p* = 0.05 and *p* = 0.006). Non‐white respondents had higher motivation than White individuals (*p* = 0.03). Those reporting higher perceived socioeconomic status reported lower mean motivation scores (*p* = 0.003). Insurance status, employment status, gender, and cancer history were not significantly associated with motivation.

**TABLE 3:** Predictors of genetic testing motivation from multivariable regression analysis (*n* = 766).

Independent variables	*B* (standard error)	*p*
Psychosocial variables		
Barrier index score	−0.07 (0.03)	0.006
Self‐efficacy	0.27 (0.08)	<0.001
Mistrust	−0.67 (0.16)	<0.001
Knowledge	−0.004 (0.02)	0.86
Age (years)		
18–50 (ref)	–	
51–70	−0.46 (0.23)	0.05
70+	−0.86 (0.25)	0.006
Race and/or Ethnicity		
White (ref)	–	–
All other races	0.53 (0.25)	0.03
Income		
Living comfortably (ref)	–	–
Not living comfortably	0.55 (0.18)	0.003
Gender		
Female (ref)	–	–
Male	−0.09 (0.18)	0.60
Non‐binary, Genderqueer or Transgender	0.13 (0.87)	0.88
Insurance		
Private (ref)	–	–
Public or no insurance	0.25 (0.20)	0.21
Employment		
Unemployed/student (ref)	–	–
Retired	−0.25 (0.37)	0.50
Working	−0.18 (0.35)	0.61
Cancer history		
Level 1 cancers (ref)	–	–
Level 2 cancers	−0.13 (0.27)	0.64
Level 3: Family history only	0.25 (0.27)	0.36

*R*	= 0.34
*R* ^2^	= 0.11
Adjusted *R* ^2^	= 0.10
*F*	= 6.44, *p* < 0.001

Among the participants with a personal history of cancer who answered the clinician autonomy support items (*n* = 480), clinician autonomy support was not significantly associated with motivation (data not shown).

## 4 DISCUSSION

In our sample of 799 participants who met guidelines for, but had not previously undergone genetic testing, we identified several correlates of motivation to get testing. In multivariable analysis, barriers and medical distrust were negatively associated with motivation to test, whereas self‐efficacy was positively associated with motivation. Previous studies have identified the assessed barriers as negative predictors for testing, while this is among the first reports identifying self‐efficacy and medical distrust as determinants of motivation for testing.

Consistent with prior literature on medical mistrust and distrust with genetic testing, we found an inverse association between healthcare distrust and genetic testing motivation. Use of the HCSDS allowed for assessment of participants' individualized views about the healthcare system, rather than their perceptions of mistrust specific to certain groups of people (e.g., gender minority group members, people with disabilities) and/or racial and ethnic groups (Rose et al., 2004; Williamson & Bigman, 2018). Practically, both an assessment of general medical distrust and race–group‐specific mistrust may be needed when intervening in these populations. Efforts to assuage distrust both at the individual and system level may be needed. Knowing the specific type of distrust, whether related to data privacy/security, the health system as a whole, or one's particular racial and/or ethnic group or community can lead to tailored interventions and counseling to better address the root causes. This may be completed through relationship building and outreach within specific communities, transparent written and verbal communication from healthcare providers about the possible outcomes, costs, risks and benefits of getting genetic testing, and improved provider understanding that decisions regarding genetic testing may take time and require ongoing support.

While the impact of barriers on genetic testing intention has been previously described in patients with a personal history of cancer, our study includes individuals that have a variety of cancer types or who may meet testing criteria based on a family history rather than a personal history of cancer (Austin et al., 2024). Identification of barriers in this population is important in direct patient care and allows for tailored intervention which can be completed through traditional genetic counseling, written educational content, videos, or patient testimonials. Intervening on barriers could include counterarguments to dispel common misconceptions related to genetic testing related to cost, inheritance, and actionability of results, as well as motivational interviewing‐type rolling with resistance (e.g., acknowledging fears, reframing; Resnicow & McMaster, 2012). Our clinical trial incorporates tailored messaging through a variety of health education techniques based on the participants' self‐reported barriers and motivating factors to get genetic testing (Gerido et al., 2023).

There were three sociodemographic variables associated with decreased motivation to get genetic testing relative to their respective reference groups: younger ages (age 18–50), White race, and those with higher perceived socioeconomic status. Selkirk et al. (2014) similarly found lower rates of testing among younger participants (59.8 vs. 52.6 years, *p* < 0.0001), although their study assessed genetic testing uptake directly, rather than motivation (Selkirk et al., 2014). In contrast to our study, other publications have found younger age to be positively associated with genetic testing uptake and/or intention in individuals with cancer (Aguiar et al., 2023; Anderson et al., 2014; Ladd et al., 2020; Turza et al., 2022).

The racial and socioeconomic findings of this study are, to the best of our knowledge, novel as most prior studies focused on uptake rather than motivation. A systematic review of sociodemographic factors associated with genetic testing completed by Willis et al. (2017) found higher income was inconsistently associated with genetic testing uptake (positive predictor in two studies, not associated in three), and African American ethnicity was associated with lower uptake of testing in one study. These inconsistent findings may be because predictors of uptake may differ from predictors of motivation. Additionally, these findings may be in part due to selection bias in our sample as only individuals who had not previously completed genetic testing were included. Individuals with higher motivation likely already completed genetic testing, leaving only the less‐motivated individuals in our study cohort.

The positive association found between self‐efficacy and motivation, while new, is not surprising given the robust literature documenting the association between self‐efficacy and health behaviors (Bandura, 1982; Lam & Lin, 2024; Langford et al., 2024; Wong, 2012). Similar to our findings, Ding et al. (2021) found that in Black women, higher self‐efficacy was associated with more positive attitudes toward genetic testing and greater confidence in laws protecting against genetic discrimination (Genetic Information Non‐Discrimination Act, GINA), while those with lower self‐efficacy had greater perceived difficulty in getting testing (Ding et al., 2021). Self‐efficacy remains an intervention target to help patients overcome and take action toward their barriers. Enhancing self‐efficacy can be completed through the provision of skills (increased knowledge about genetic testing purpose, cost, and process) and cognitive restructuring.

Limitations of this study include lack of diversity in our sample: the majority of participants were White and the generalizability of our findings to more diverse clinical populations merits further examination. Additionally, given the small sample size for non‐White individuals, these individual racial and/or ethnic groups could not be disaggregated in the analysis. Our study, which was a randomized intervention trial, only included individuals who had not previously undergone genetic testing. It is possible that this resulted in selection bias and that individuals who had previously undergone genetic testing might experience different psychosocial motivators and barriers to genetic testing that are not captured in this study. Our study measured only motivation to undergo genetic testing rather than actual uptake of genetic testing. Since our study was cross‐sectional in design, longitudinal prediction of testing behavior is not possible, and longitudinal studies to examine causal pathways are needed. As part of a larger clinical trial, we plan to assess the relationship between these psychosocial factors and actual genetic testing uptake for individuals who have not previously undergone such testing.

Our study has identified several intervention targets, both demographic and psychosocial, that could inform both group and individual level interventions for genetic testing. Our future studies include assessing how these psychosocial factors, barriers, and motivators predict genetic testing uptake rather than motivation.

## AUTHOR CONTRIBUTIONS

Sarah Austin and Kenneth Resnicow conceptualized the study. All authors contributed to the data acquisition for this study. Sarah Austin and Emerson Delacroix completed data analysis, with input and supervision from John D. Rice and Kenneth Resnicow. Sarah Austin wrote the first draft of the manuscript. All authors critically revised the manuscript for intellectual content. Sarah Austin confirms that she had full access to all the data in the study and takes responsibility for the integrity of the data and the accuracy of the data analysis. All authors gave final approval of this version to be published and agreed to be accountable for all aspects of the work in ensuring that questions related to the accuracy or integrity of any part of the work are appropriately investigated and resolved.

## CONFLICT OF INTEREST STATEMENT

Authors Sarah Austin, Emerson Delacroix, John D. Rice, Erika Koeppe, Elena M. Stoffel, Jennifer J. Griggs, and Ken Resnicow declare that they have no conflict of interest.

## ETHICS STATEMENT

The University of Michigan Medical School Institutional Review Board (IRB) approved this study under HUM00192898. Informed consent was obtained for all participants included in this analysis.

## FUNDING STATEMENT

Funding source: National Cancer Institute, Grant/Award Number: U01 CA232827. Innovative Approaches to Expand Cancer Genetic Screening and Testing for Patients and Families in a Statewide Oncology Network through Community, State, and Payer Partnerships (PI Stoffel, Griggs, Resnicow) and National Cancer Institutes of Health under Award Number P30CA046592 by the use of the Rogel Cancer Center Health Communications Shared Resource: Center for Health Communications Research.

## CLINICAL TRIAL REGISTRATION

This study is registered at ClinicalTrials.gov (NCT05162846).

## Supporting information


Tables

## Data Availability

The dataset supporting this study is available at https://deepblue.lib.umich.edu/data/concern/data_sets/5m60qs76r. Delacroix, E., Austin, S., Bacon, E., Rice, J., Hanson, E. N., Stoffel, E. M., Roberts, S., Ulhmann, W., Griggs, J. J., Resnicow, K. *MiGHT Baseline Survey Data* [Data set], University of Michigan ‐ Deep Blue Data. DOI pending as of 2025–03–10 T14:30:36–04:00.
