# Supplementary material for: Psychosocial predictors of hereditary cancer genetic testing motivation in untested individuals
Source: J Genet Couns. 2025 Oct 15;34(5):e70122. doi: 10.1002/jgc4.70122 (PMC12528973; doi:10.1002/jgc4.70122)
Supplement: Supplementary file 1 — Tables [file JGC4-34-0-s001.docx]

| **Barrier Items** | **Correlation with motivation** | **P-value** | **Mean score** | **Std. Deviation** |
| --- | --- | --- | --- | --- |
| Knowing my genetic status would not change… | | | | |
| ...my cancer treatment. | -0.040 | 0.266 | 3.01 | 1.313 |
| ...my cancer follow-up care and monitoring. | 0.014 | 0.705 | 3.01 | 1.422 |
| ...what I can do to prevent getting another cancer. | -0.005 | 0.897 | 2.86 | 1.409 |
| Genetic testing would not help me deal with my fears about having my cancer come back or getting a new cancer. | **-0.295** | **<0.001** | 2.40 | 1.020 |
| I have too much going on with my current cancer diagnosis to think about genetic testing right now. | **-0.388** | **<0.001** | 1.88 | 0.849 |
| I am too overwhelmed to think about genetic testing right now. | **-0.303** | **<0.001** | 1.82 | 0.830 |
| I don't have time to get genetic testing. | **-0.351** | **<0.001** | 1.76 | 0.794 |
| I would worry about how genetic testing would affect my health insurance rates. | **-.0168** | **<0.001** | 2.87 | 1.268 |
| If I were found to carry an altered gene, I worry it would affect my eligibility for health insurance (e.g. be considered a pre-existing condition). | **-0.148** | **<0.001** | 2.87 | 1.259 |
| If I were found to carry an altered gene, I would worry about who would have access to my test results. | **-0.180** | **<0.001** | 2.37 | 1.185 |
| If I were found to carry an altered gene, I worry it would affect my life insurance policy. | **-0.111** | **0.002** | 2.66 | 1.220 |
| Getting genetic testing would cost me too much money. | **-.127** | **<0.001** | 2.88 | 1.063 |
| I worry that my health insurance would not cover the cost of genetic testing. | **-0.096** | **0.007** | 3.32 | 1.192 |
| If I was found to have an altered gene, I would feel… | | | | |
| ...angry | **-0.073** | **0.042** | 2.36 | 1.005 |
| …hopeless/sad | **-0.110** | **0.002** | 2.49 | 1.016 |
| …guilty about passing it on | 0.041 | 0.256 | 2.77 | 1.213 |
| ...anxious | **-0.079** | **0.028** | 3.25 | 1.108 |
| I would feel anxious while waiting for my results. | **-0.071** | **0.048** | 2.91 | 1.125 |
| Knowing that I carry an altered gene would make me feel defective. | **-0.118** | **<0.001** | 1.93 | 0.891 |
| I’m not sure if the test is accurate. | **-0.229** | **<0.001** | 2.27 | 0.864 |
| I don’t know how genetic testing benefits me. | **-0.403** | **<0.001** | 2.39 | 1.021 |
| I don’t know how to get genetic testing. | -0.062 | 0.084 | 3.15 | 1.087 |
| Genetic tests can be used to make certain racial and ethnic groups seem inferior. | **-0.147** | **<0.001** | 2.49 | 1.105 |

Supplementary Table 2. Internal Consistency Alpha for Psychosocial Items

| **Measure** | **Scoring Method** | **Alpha coefficient** |
| --- | --- | --- |
| Healthcare distrust (10 items) | Mean | 0.76 |
| Self-efficacy (6 items) | Mean | 0.84 |
| Genetic knowledge/literacy (16 items) | Number correct | 0.81 |
| Clinician autonomy support (7 items) | Mean | 0.94 |
| Barriers | Count of 4’s and 5’s to create index score | N/A |
| Motivation score | Mean | 0.86 |
